# Minimum Intervention Oral Care (MIOC) - Overcoming Implementation Barriers: An International Expert Consensus

**DOI:** 10.1016/j.identj.2026.109532

**Published:** 2026-04-01

**Authors:** Falk Schwendicke, Avijit Banerjee, Sarah R. Baker, Martha Büttner, Laura Ceballos, Maximiliano S. Cenci, Sophie Doméjean, Margherita Fontana, Sevil Gurgan, Niek J.M. Opdam, Tamara Perić, Ruth M. Santamaría, Nicola Scotti, Livia M.A. Tenuta, Lezize Sebnem Turkun, Ivana Miletić

**Affiliations:** a Department of Conservative Dentistry, Periodontology and Digital Dentistry, LMU University Hospital, Munich, Germany; b Research Centre of Oral Clinical Translational Sciences, Department of Conservative & MI Dentistry, Faculty of Dentistry, Oral & Craniofacial Sciences, King’s College London, London, UK; c Institute of Rural and Coastal Health, College of Health and Science, University of Lincoln, Lincoln, UK; d Dampsoft GmbH, Damp, Germany; e Department of Nursing and Stomatology, Health Sciences Faculty, Rey Juan Carlos University, Alcorcón, Madrid, Spain; f Department of Dentistry, Research Institute for Medical Innovations, Radboud University Medical Center, Nijmegen, The Netherlands; g Department of Restorative Dentistry and Endodontics, Clermont-Ferrand, France; h Estaing Hospital Clermont-Ferrand, Dental Service, Clermont-Ferrand, France; i Department of Cariology, Restorative Sciences and Endodontics, University of Michigan, Ann Arbor, Michigan, USA; j Department of Restorative Dentistry, Faculty of Dentistry, Hacettepe University, Ankara, Turkey; k Department of Dentistry, Radboud University Medical Centre, Nijmegen, The Netherlands; l Clinic of Pediatric and Preventive Dentistry, School of Dental Medicine, University of Belgrade, Belgrade, Serbia; m Department of Pediatric Dentistry, Medical University of Greifswald, Greifswald, Germany; n Department of Surgical Sciences, Dental School Lingotto, University of Turin, Turin, Italy; o Department of Restorative Dentistry, Ege University, Izmir, Turkey; p Department of Endodontics and Restorative Dentistry, School of Dental Medicine, University of Zagreb, Zagreb, Croatia

**Keywords:** Caries, Health policy, Minimum intervention dentistry, Prevention, Remuneration, Sustainable dentistry

## Abstract

**Objectives:**

The minimum intervention oral health care (MIOC) delivery framework shifts restorative dentistry from a predominantly operative, procedure-centred model towards a person-focused, prevention-oriented and tooth-preserving approach. This international consensus aimed to develop agreed, evidence-informed recommendations to support uptake of MIOC.

**Methods:**

A structured expert consensus process was conducted. Sixteen international experts participated in a structured in-person meeting and consented on fourteen recommendations, all with 100% agreement.

**Results:**

Implementation of MIOC requires alignment of reimbursement systems, quality indicators, coding structures and digital infrastructures with preventive, diagnostic and active surveillance-based care pathways. Recommendations emphasise recognition and reimbursement of risk assessment and supportive care, integration of team-based delivery models, embedding MIOC competencies across undergraduate and postgraduate education, and ensuring that digital and AI technologies demonstrate person-centred benefit consistent with MIOC principles.

**Conclusions:**

The consensus provides a coordinated framework to support implementation and advance equitable, sustainable and prevention-oriented oral health care delivery globally.

## Introduction

The minimum intervention oral health care (MIOC) delivery framework represents a fundamental shift in oral health care delivery, reframing restorative dentistry from a predominantly operatively focused ‘treatment of disease’ discipline, towards a holistic, salutogenic^1^ ‘promotion of health – what matters to you?’ model of care. It emphasises early lesion detection and disease/pathology diagnosis; personalised risk and susceptibility-based preventive management; tooth-preserving treatments when needed; and long-term team-delivered review with supportive care, using active surveillance.^2^^,^^3^ Although rooted in cariology and preventive sciences, MIOC also applies in medicine with interprofessional education and collaborative practise, as well as to other clinical disciplines in dentistry (including cariology, periodontology, and tooth wear management). It integrates patient behaviour change management with clinical nonoperative, microinvasive and minimally invasive interventions, alongside structured diagnostics, staged personalised care planning, active surveillance with supportive care, and person-focused, shared decision-making across the life course, to facilitate the patient’s maintenance of lifelong oral and dental health. It implements the current evidence-based literature regarding modern caries care and management^4^^,^^5^ and is based on four broad, interlinking clinical domains (Figure):

• Identify: methods of detection/diagnosis/prognosis of oral disease – risk/susceptibility assessment of the patient, use of investigations to formulate staged personalised care plans,
• Prevent lesions and control disease: nonoperative and/or micro-invasive primary and secondary prevention of diagnosed conditions,
• Minimally invasive (MI) operative intervention/MI dentistry: tooth-preserving operative (surgical) treatments to repair or replace tissue damage/loss (tertiary prevention), and
• Re-assess: review/maintenance/recall of patient behaviours, treatment outcomes, including the advice/care offered by the dentist and the oral health care team. Active surveillance of early lesions, tooth-restoration complexes, susceptibility to disease, and avoiding unnecessary interventions, including replacing functional restorations.^6^

**Fig:**
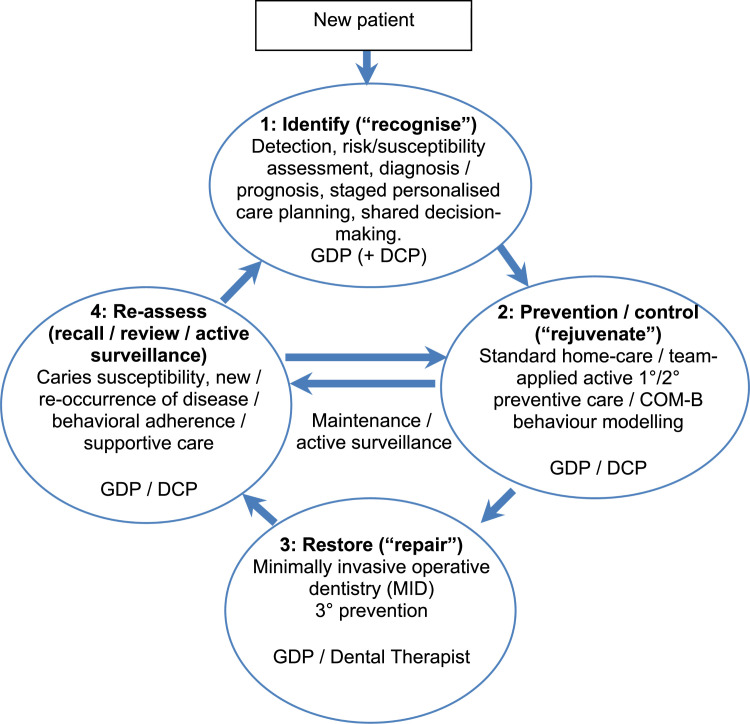
The person-focused minimum intervention oral care (MIOC) delivery framework showing the four related clinical domains of *identify -* patient assessment/ diagnosis; non-operative *prevention* of lesions or *control* of disease; *minimally invasive* operative intervention; and *re-assess* (review/ recall/ active surveillance). The arrows indicate the direction of the patient journey through this care delivery framework and within each domain an indication is given of the members of the oral health care team who might be involved (GDP – general dental practitioner; DCP – dental care professionals [includes oral health educators, extended duties dental nurses, dental hygienists, dental therapists, practise managers, clinical dental technicians, reception staff]).^2^

With regard to dental caries management, evidence consistently demonstrates that early carious lesions can be arrested or reversed,^7^, ^8^, ^9^, ^10^ that conservative management preserves tooth structure and pulp sensibility, and that repeated invasive operative intervention accelerates the destructive dental-restorative cycle, increasing biological cost, patient burden, and long-term health system expenditure.^11^, ^12^, ^13^ Beyond clinical outcomes, MIOC aligns with broader health system goals, including planetary health and One Health,^14^ equity in access, and salutogenic value-based oral health promotion,^15^ as reflected in international clinical guidelines and educational frameworks.^3^^,^^7^^,^^16^, ^17^, ^18^, ^19^, ^20^, ^21^, ^22^, ^23^, ^24^, ^25^, ^26^, ^27^, ^28^, ^29^, ^30^, ^31^, ^32^, ^33^, ^34^, ^35^, ^36^, ^37^, ^38^, ^39^, ^40^ MIOC fulfils the strategic objective of the World Health Organisation’s Bangkok Declaration on oral health and the environment, where it ‘promotes preventive, less invasive, climate resilient, environmentally sustainable and safe oral health care by adopting mercury free and eco-friendly products, minimizing the use of single-use plastics and nonbiodegradable materials, managing waste responsibly, using natural resources efficiently, and reducing carbon emissions.’^41^

Despite this evidence base, implementation of MIOC protocols in routine clinical primary care practise remains inconsistent.^25^^,^^42^^,^^43^ In many health care systems, operative and procedure-centred paradigms continue to dominate clinical pathways, reimbursement mechanisms, educational training, assessment and professional identity.^44^^,^^45^ Core components of MIOC, such as risk/susceptibility assessment, lesion activity grading, supportive care with longitudinal active surveillance, are frequently under-recognised, inadequately coded and therefore inadequately reimbursed.^46^^,^^47^ Workforce structures, scopes of practise of oral health care team members,^32^^,^^48^ digital infrastructures and regulatory frameworks are often misaligned with preventive and longitudinal health care models. Consequently, MIOC is commonly delivered as an exception, rather than as the default standard of oral health care.

This persistent evidence–practise gap reflects a complex interaction of educational, cultural, organisational, financial, regulatory and technological barriers. Addressing these barriers requires coordinated, system-level change across multiple stakeholders, including clinicians, educators, professional and patient bodies, funders, regulators and policymakers.^49^^,^^50^ Consensus-based guidance is therefore needed to articulate shared principles and priorities that can support implementation while remaining adaptable to diverse national and local contexts.

The aim of this international consensus was to develop agreed, evidence-informed recommendations to support the uptake of MIOC across wider clinical practise, education, workforce organisation, and higher-level health system design, informed by an analysis of key implementation barriers highlighted in dental caries management.

## Methods

### Overall approach and study design

This work employed a structured, evidence-informed expert consensus process to identify barriers to the implementation of MIOC for caries management and to develop agreed recommendations to improve its uptake across clinical practise, education, workforce organisation and health system design. The methodological approach was informed by established consensus and Delphi-type processes^51^ and closely followed procedures previously used in international oral health consensus work, including predefined agreement thresholds, structured discussion and anonymous voting.^52^

### Steering group and project initiation

The steering group (FS, AB, and IM) initiated and coordinated the project. It defined the scope and objectives of the consensus, agreed on the methodological approach, identified key domains relevant to MIOC implementation and oversaw the preparation of background evidence. It was agreed that a consensus process supported by narrative evidence synthesis would be most appropriate to address the complexity and system-level nature of MIOC implementation.

### Definition of scope and domains

During the preparatory phase, four key domains were identified as central to understanding and addressing barriers to MIOC implementation:

1. Education and training
2. Workforce and practise organisation
3. Psychological and cultural barriers, including professional identity
4. System-level, financing, regulatory, governance and technological barriers

These domains guided the commissioning of background reviews, the structure of discussions during the consensus meeting and the organisation of the resulting consensus statements.

### Evidence-based and preparatory reviews

To inform the consensus process, narrative (nonsystematic) reviews were prepared for each of the four domains by the steering group. These reviews synthesised available scientific literature, policy documents, international guidance and illustrative examples related to MIOC, minimally invasive dentistry (MID), diagnostics and active surveillance, educational frameworks, workforce models, reimbursement systems, regulation and digital and AI-enabled technologies. The purpose of these reviews was not to provide exhaustive evidence synthesis, but to establish a shared knowledge base, identify recurring themes and gaps, all to aid and support informed discussion among the consensus participants. All review materials were circulated to participants in advance of the consensus meeting. Although these were intended as a basis for discussion, they were not chosen to provoke controversy; rather, they focused on well-established findings and aimed to establish consensus.

### Consensus panel and participant selection

The consensus panel consisted of 16 international experts with recognised expertise in cariology, MID, dental public health, health services research, education, industry and policy. Participants were selected by the steering group to ensure diversity in disciplinary background and international perspectives. All participants had prior experience with MIOC or closely related professional disciplines.

### Consensus meeting and statement development

The consensus process was conducted during a structured in-person meeting, hosted on 1-2 December 2025 in Munich, Germany. The meeting began with presentations summarising the aims of the project, the scope of the consensus and the findings of the narrative reviews. These presentations were followed by extended plenary and group discussions; participants were divided into three groups themed ‘System & Financing’, ‘Workforce & Diagnostics’, and ‘Education and Culture’. Each of them discussed 5 to 7 of the original 18 recommendations drafted by the steering group. Groups were tasked with discussing the recommendations and making suggestions to reflect shared positions on key principles and actions needed to support MIOC implementation. The statements were intentionally phrased to be principle-based and internationally applicable, while allowing for contextual adaptation to different global health care systems. Participants reconvened for a general discussion during which discussion groups shared their suggestions to the original recommendations (modifying, merging), which were actively discussed by all participants. The final 14 recommendations were subjected to anonymous online voting.

### Voting procedure and consensus rules

Consensus was assessed using an anonymous voting procedure. Participants voted on each statement using a binary response format (‘yes’ or ‘no’), with the option to abstain. Abstentions were considered as nonagreement for the purpose of determining consensus.

Before voting commenced, the rules of the voting process were explicitly agreed upon by all participants. A priori, a statement was considered to have achieved consensus if at least 75% of all votes indicated agreement. Two rounds of voting were planned to allow discussion and revision of statements that did not reach the predefined threshold in the first round.

Voting was conducted using an electronic polling system (Mentimeter 4), allowing participants to vote independently and without influence from other panel members. Participants were also allowed to provide qualitative comments on individual statements. All statements presented in this paper reached full consensus (100% agreement) in the first round of voting.

### Reporting and funding

The conduct and reporting of the consensus process adhered to established guidance for the conduct and reporting of Delphi and expert consensus studies.^51^ The methods were designed to ensure transparency regarding scope definition, evidence inputs, participant selection, voting procedures and agreement thresholds.

The consensus meeting, including travel expenses for the participants, was supported independently by the International Society of Minimum Intervention Dentistry (MIS).

## Results

The final Delphi consensus resulted in 14 recommendations, organised thematically from overarching principles to system-level, workforce, educational and cultural enablers.

### MIOC as the default standard of care delivery

Recommendation 1 – MIOC framework should be the standard of care

Statement

**MIOC should be adopted as the default evidence-based approach for promoting oral health (eg, for dental caries, emphasising early detection, prevention/control, tooth preservation and supportive care with active surveillance).**

*Explanation*

This statement establishes MIOC as the normative delivery framework for oral health care and caries management, with MI operative interventions reserved for situations where preventive, nonoperative or microinvasive strategies are insufficient on their own, to manage existing carious lesions.

### System structures, quality indicators, policy alignment

Recommendation 2 – System structures should support MIOC

Statement

**Staged clinical pathways, reimbursement structures and quality indicators should be redesigned so that MIOC implementation is routine rather than exceptional.**

*Explanation*

Quality indicators should move beyond counting procedures towards outcomes such as caries prevention and arrest, levels of restoration replacement/longevity, maintenance of pulp sensibility, improvements in oral health-related quality of life, care equity, cost-effectiveness and environmental impact/ sustainability. Governance from health authorities, regulators, health technology assessment bodies and funders should incorporate these outcomes when shaping national policies and coverage decisions.

### Ontologies, coding, data infrastructure

Recommendation 3 – Ontologies and coding systems should appropriately reflect the MIOC clinical domains.

Statement

**Ontologies, diagnostic and procedural coding systems should explicitly include team-delivered preventive, nonoperative, microinvasive treatments and active surveillance/ supporting care activities.**

*Explanation*

Harmonisation of coding systems - within and across countries, electronic health records and software platforms - is required across the whole of health care. Oral and dental diagnostic and procedural codes should be defined and utilised, reflecting frameworks such as the Core Cariology Curriculum (currently being updated),^53^^,^^54^ and adapted locally as required. As artificial intelligence (AI) and detection technologies continue to evolve, diagnostic codes derived from validated, accurate, automated data capture may be required.

### Diagnostics, supportive care, active surveillance

Recommendation 4 – Diagnostics and supportive care should be reimbursed as essential care.

Statement

**Risk/susceptibility assessment, lesion activity grading and supportive care should be reimbursed as core components of patient management.**

*Explanation*

Supportive care should be defined for different age and susceptibility groups. Emerging technologies that allow collected clinical data triangulation may allow more objective measurement of active surveillance health-promoting outcomes, but also raise issues related to cost, access, workforce impact and overdiagnosis/overtreatment.

Recommendation 5 – Diagnostics and active surveillance should be the gatekeepers of intervention.

Statement

**Routine care should include standardised diagnostic and active surveillance protocols to determine whether, when and how to intervene in dental caries management.**

*Explanation*

Standardised diagnostic and active surveillance protocols provide not only consistency across different clinical teams but also enable safer and easier sharing of tasks between team members. Standardised protocols reduce the need for supervision and allow widening of the scope of practise across oral health care team members, thereby maintaining quality of care.

### Reimbursement, incentives

Recommendation 6 – Reimbursement systems should reward preventive, nonoperative and microinvasive care for patients with evidence of increased disease susceptibility

Statement

**Reimbursement systems should reward preventive, nonoperative and microinvasive care for patients with evidence of increased disease susceptibility, across the life course.**

*Explanation*

Current dental reimbursement systems are predominantly procedure-based and reward operative interventions rather than preventive, nonoperative/microinvasive care. Preventive care for children, adolescents, adults and high-susceptibility patients is often limited or insufficiently reimbursed, creating disincentives to actioning the prevention-focused, oral health-promoting MIOC principles.

### Digital and AI technologies

Recommendation 7 – Digital and AI technologies should demonstrate benefits.

Statement

**Digital and AI technologies should demonstrate benefits for oral health care provision according to the MIOC framework.**

*Explanation*

Further research is needed to determine whether new digital and AI technologies deliver measurable person-focused clinical benefits aligned with the MIOC goals, rather than merely improving technical efficiency, diagnostic yield or throughput.

### Workforce, scope of practice, and care settings

Recommendation 8 – MIOC includes a range of activities and team members.

Statement

**MIOC should include a range of activities that can be delivered by different members of the oral health care team in a coordinated approach.**

*Explanation*

Scope-of-practice regulations vary nationally and must be aligned with workforce planning to enable effective task-sharing. This should be aligned to primary (including community settings), secondary and tertiary care services, in order to achieve equity in care access.

Recommendation 9 – Care must be provided in different settings beyond the dental surgery.

Statement

**To deliver MIOC equitably, care should be provided in different community settings beyond the dental surgery/clinic.**

*Explanation*

Underserved and high needs populations should be targeted through community-level delivery of MIOC, including within schools, community centres, long-term care facilities and in rural or underserved areas. There are examples where some nations have prioritised this approach in their health care delivery planning.^55^

### Education and professional development

Recommendation 10 – MIOC clinical competencies should be embedded into curricula and accreditation standards, both at the under- and postgraduate levels.

Statement

**Accreditation agencies, dental schools or training facilities, and dental educational programmes should require competencies to be aligned with MIOC principles across all dental disciplines.**

*Explanation*

Oral and dental education should move from operative quotas towards competency-based and experiential assessment. It should reflect current epidemiology and the oral health care needs of the served patients and populations. The MIOC competencies/ principles and clinical domains (Figure) should underpin the content of oral and dental health curricula across each of the team roles and across the restorative disciplines.

Recommendation 11 – Faculty staff development should support MIOC education.

Statement

**Educational institutions should provide structured faculty and staff development aligned with the MIOC principles.**

*Explanation*

Faculty development is a prerequisite for adapting curricula and harmonising teaching in line with MIOC principles, as consistent implementation requires coordinated and consistent understanding, messaging and application across different dental disciplines. Even though the MIOC clinical domains are stable, the clinical content within them will adapt and change as developmental progress is made in the professional understanding and management of behavioural psychology, technology and clinical implementation.

Recommendation 12 – MIOC competencies of oral health care team members should be updated.

Statement

**MIOC competencies of oral health care team members should be updated on an ongoing basis.**

*Explanation*

Core clinical competencies require national mandatory review as deemed by regulators and updating as part of continuing professional development in several health care systems around the world. There is a need for these MIOC competencies within the four clinical domains to be included in mandatory continuing professional development requirements for possible professional re-validation and to ensure optimal patient care is maintained.

### Clinical systems, professional identity

Recommendation 13 – Patient management systems and electronic health records should support MIOC delivery.

Statement

**Clinical recording systems should support risk/susceptibility assessment, early lesion detection, lesion staging and grading documentation and preventive/non-operative care pathways, delivered by oral health care team members.**

*Explanation*

Clinical recording systems should enable structured documentation of individual susceptibility, early carious lesion detection, including extent and activity status (staging and grading),^56^ and preventive nonoperative or microinvasive care pathways, rather than focusing primarily on operative procedures. Such functionality is essential to support consistent implementation, review and evaluation of MIOC-aligned care.

Recommendation 14 – Professional identity and patient expectations should be reframed according to MIOC principles.

Statement

**Professional identity and patient expectations should be reframed according to MIOC principles.**

*Explanation*

Disease control (e.g. dental caries, periodontal disease) along with supportive care, including active surveillance, should be recognised as high-skill clinical practises across oral health care team members. Patients should be educated and informed that effective, long-term oral health care does not always require operative intervention. They should be informed of the evidence supporting other less invasive interventions and that modest personal behaviour change can yield substantial oral/dental and systemic health benefits across the life-course.

## Discussion

The Delphi consensus process, including the assimilation of updated evidence and the recommendations presented, highlights that the limited implementation of MIOC is not a problem of insufficient scientific or clinical evidence, but due to misalignment between knowledge or understanding, professional practise and health system structures. Despite overall agreement on the clinical, public health and economic value of the MIOC framework, implementation remains constrained by educational traditions, legacy reimbursement and regulatory frameworks, workforce organisation, incoherent digital infrastructure and entrenched professional and patient expectations. The recommendations developed through this consensus, therefore, address not only clinical practise but the wider ecosystem in which oral health care is delivered.

For policymakers, regulators and funders, this consensus provides a structured framework to support alignment of oral and dental health care systems, with contemporary evidence. The recommendations emphasise a shift away from invasive procedure-centred ‘treatment of disease’ models towards holistic, salutogenic, ‘optimising health’ outcome-oriented approaches that reward oral and dental health promotion, disease prevention or control, and tooth-preserving treatments where justified. They can be used to inform the redesign of benefit packages, reimbursement schedules and quality indicators. Health technology assessment bodies and regulatory agencies may also draw on this consensus when defining evaluation criteria for new interventions and technologies, ensuring that approvals and coverage decisions reflect the MIOC principles rather than artificially validated invasive procedural endpoints. At a system level, the recommendations support reforms that enable task-sharing, expand the scope of practise of members of the oral health care team (dental nurses, dental hygienists, dental therapists, clinical dental technicians, practice managers, reception staff) where appropriate, and facilitate delivery of MIOC in primary care community settings, particularly for underserved and high-susceptibility populations.^57^

For educators and accreditation bodies, the consensus articulates a clear mandate to embed MIOC competencies across dental curricula and disciplines. The call to move from operative quotas towards competency-based experiential assessment directly addresses one of the most persistent barriers to implementation. Accreditation agencies may use these recommendations to update standards and learning outcomes, while dental schools and training programmes can use them to justify curriculum reform, investment in faculty development and redesign of clinical assessment systems.^58^ Aligning education with the real and ever-changing epidemiologic landscape is essential to ensure that training reflects real-world needs rather than historically dominant invasive treatment models.^59^

For clinicians and oral health care teams, the consensus reframes MIOC not as a reduction in care, but as a highly-skilled, evidence-based approach to disease management and delivering better oral health. The emphasis on early detection, diagnostics, nonoperative and/or microinvasive interventions for prevention and active surveillance underscores their role as the foundation of clinical shared decision-making and supports the integration of preventive, nonoperative and microinvasive strategies into routine care. By explicitly recognising that MIOC comprises activities deliverable by different members of the oral health care team, the recommendations support coordinated, team-based care models that can improve access, efficiency and continuity of preventive and supportive services. The consensus also provides clinicians with a framework to engage patients in shared decision-making and to recalibrate expectations and values around what constitutes effective oral health care.

For researchers, this consensus identifies clear priorities for implementation science^60^ and oral health services research.^61^ These include the development, triangulation of clinical data and validation of MIOC-aligned quality indicators, evaluation of reimbursement and incentive reforms, assessment of workforce and scope-of-practise models and the investigation of digital and AI technologies within preventive and active surveillance-based staged personalised care pathways. Longitudinal and pragmatic evaluations are particularly needed to generate evidence on outcomes such as early disease arrest, restoration avoidance, patient-reported outcomes, equity of care delivery and access, environmental impact and cost-effectiveness to the health care system. Data is also required to explore how the MIOC framework can be adapted and scaled across diverse health system contexts, including low-resource settings.

To foster uptake and dissemination, the consensus recommendations should be translated into targeted outputs for different audiences, such as policy briefs, educational guidance and clinical implementation technologies. Engagement with professional associations, regulators, payers, accreditation bodies and patient organisations will be critical to promote awareness, legitimacy, and ultimate adoption. Pilot implementation projects, supported by appropriate funding and evaluation frameworks, may help demonstrate feasibility and impact in real-world settings. Integration of such MIOC principles into national clinical guidelines, reimbursement schedules and digital health infrastructures represents a key next step towards sustainable adoption.

Some limitations of this consensus should be acknowledged. The recommendations were informed by narrative rather than systematic reviews. Although the preparatory syntheses drew on contemporary scientific and policy literature, they did not follow predefined search protocols or formal evidence grading, and incomplete evidence capture cannot be excluded. Future targeted systematic reviews could strengthen the evidentiary basis of specific recommendations. The panel, while appropriate for a structured expert consensus and comprising internationally recognised experts across multiple disciplines, did not cover all relevant participants, including patients, frontline practitioners outside academic settings, payers and policymakers from low- and middle-income countries. As many recommendations address system-level reform, broader stakeholder engagement would enhance future iterations. The statements are intentionally principle-based and therefore require contextual adaptation before implementation at the national or local level. Finally, while consensus indicates agreement among experts, and reflects the principles expressed in most of the current clinical practise guidelines,^62^^,^^63^ it does not constitute empirical validation. The real-world impact, feasibility, cost implications and potential unintended consequences of implementing these recommendations require rigorous evaluation through implementation and health services research across diverse contexts.

## Conclusion

In conclusion, this consensus provides a comprehensive, multilevel framework of recommendations to address the persistent gap between evidence and practise of the MIOC delivery framework. By aligning policy, education, workforce organisation, technology and professional culture with such MIOC principles, the recommendations offer a pathway towards more equitable, sustainable and evidence-based oral health care delivery for all. The challenge now lies in translating consensus into coordinated action.

## Author contributions

*Contributed to the concept or design of this work, its conduct, analysis and interpretation, and wrote or revised the manuscript*: All authors. *Agree to be accountable for it*: All authors.

## Funding

The consensus workshop was funded by the MIS, which was an independent observer without any input to the consensus process.

## Conflict of interest

None disclosed.

## References

[1] Fisher J., Özcan M., Krejci I., Banerjee A. (2026). Equity and integration; why the oral healthcare community urgently needs to reflect on its approach to caries management. Int J Equity Health.

[2] Banerjee A. (2024).

[3] Leal S.C., Damé-Teixeira N., Brito C. (2022). Minimum intervention oral care – defining the future of caries management. Braz Oral Res.

[4] Ismail A.I., Pitts N.B., Tellez M. (2015). The International Caries Classification and Management System (ICCMS™): an example of a caries management pathway. BMC Oral Health.

[5] Pitts N.B., Banerjee A., Mazevet M., Goffin G., Martignon S. (2021). From “ICDAS” to “CariesCare International”: the 20-year journey building international consensus to take caries evidence into clinical practice. Br Dent J.

[6] Martins C., Godycki-Cwirko M., Heleno B., Brodersen J. (2019). Quaternary prevention: an evidence-based concept aiming to protect patients from medical harm. Br J Gen Pract.

[7] Urquhart O., Tampi M.P., Pilcher L. (2019). Nonrestorative treatments for caries: systematic review and network meta-analysis. J Dent Res.

[8] Schmoeckel J., Gorseta K., Splieth C.H., Juric H. (2020). How to intervene in the caries process: early childhood caries - a systematic review. Caries Res.

[9] Cabalén M.B., Molina G.F., Bono A., Burrow MF. (2022). Nonrestorative caries treatment: a systematic review update. Int Dent J.

[10] Dhanapriyanka M., Kosgallana S., Kanthi RDFC (2024). Professionally applied fluorides for preventing and arresting dental caries in low- and middle-income countries: systematic review. J Public Health Dent.

[11] Schwendicke F., Meyer-Lueckel M., Stolpe M., Dörfer C.E., Paris S. (2014). Costs and effectiveness of treatment alternatives for proximal caries lesions. PLoS One.

[12] Schwendicke F., Krois J., Robertson M., Splieth C., Santamaria R., Innes N. (2019). Cost-effectiveness of the hall technique in a randomized trial. J Dent Res.

[13] Schwendicke F., Krois J., Splieth C.H. (2018). Cost-effectiveness of managing cavitated primary molar caries lesions: a randomized trial in Germany. J Dent.

[14] Fisher J., Splieth C., Matanhire-Zihanzu C. (2024). Advancing the concept of global oral health to strengthen actions for planetary health and One Health. Int J Equity Health.

[15] Vujicic M., David G. (2023). Value-based care in dentistry: is the future here?. J Am Dent Assoc.

[16] Slayton R.L., Urquhart O., Araujo M.W.B. (2018). Evidence-based clinical practice guideline on nonrestorative treatments for carious lesions: a report from the American Dental Association. J Am Dent Assoc.

[17] Dhar V., Pilcher L., Fontana M. (2023). Evidence-based clinical practice guideline on restorative treatments for caries lesions: a report from the American Dental Association. J Am Dent Assoc.

[18] Pilcher L., Pahlke S., Urquhart O. (2023). Direct materials for restoring caries lesions: systematic review and meta-analysis-a report of the American Dental Association Council on Scientific Affairs. J Am Dent Assoc..

[19] Digmayer Romero V.H., Signori C., Uehara J.L.S. (2024). Diagnostic strategies for restorations management: a 70-month RCT. J Dent Res.

[20] Banerjee A. (2017). Implementing minimum intervention (MI) oral healthcare delivery – overcoming the hurdles. Prim Dent J.

[21] Doméjean S., Banerjee A., Featherstone JDB. (2017). Caries risk /susceptibility assessment: its value in minimum intervention oral healthcare. Br Dent J.

[22] Pitts N.B., Mazevet M.E., Mayne C. (2018). Shaping the future of dental education: caries as a case-study. Eur J Dent Ed.

[23] Schwendicke F., Splieth C., Breschi L. (2019). When to intervene in the caries process? An expert Delphi consensus statement. Clin Oral Invest.

[24] Martignon S., Pitts N.B., Goffin G. (2019). Caries care practice guide: consensus on evidence into practice. Br Dent J.

[25] Chana P., Orlans M.C, O’Toole S., Doméjean S., Movahedi S., Banerjee A. (2019). Restorative intervention thresholds and treatment decisions of general dental practitioners in London. Br Dent J.

[26] Banerjee A. (2020). Minimum intervention oral healthcare delivery - is there consensus?. Br Dent J.

[27] Banerjee A., Splieth C., Breschi L. (2020). When to intervene in the caries process? A Delphi consensus statement. Br Dent J.

[28] AlKhalaf R., Neves A., Banerjee A., Hosey MT. (2020). “MI” judgement calls: managing compromised first permanent molars in children. Br Dent J.

[29] Schwendicke F., Splieth C.H., Bottenberg P. (2020). How to intervene in the caries process in adults? Proximal and secondary caries. An EFCD-ORCA-DGZ expert Delphi consensus statement. Clin Oral Invest.

[30] Heidari E., Newton J.T., Banerjee A. (2020). Minimum intervention oral healthcare for people with dental phobia: a patient management pathway. Br Dent J.

[31] Dawett B., Deery C., Banerjee A., Papaioannou D., Marshman Z. (2022). A scoping literature review on minimum intervention dentistry for children with dental caries. Br Dent J.

[32] Young S., Dawett B., Gallie A., Banerjee A., Deery C. (2022). Minimum intervention oral care delivery for children – developing the oral healthcare team. Dent Update.

[33] Wambier D.S., Chibinski A.C.R., Wambier L.M., de Lima Navarro M.F., Banerjee A. (2023). Minimum intervention oral care management of early childhood caries: a 17-year follow-up case report. Eur J Paed Dent.

[34] Heidari E., Banerjee A., Newton JT. (2023). Feasibility of minimum intervention oral care delivery for individuals with dental phobia. BMC Oral Health.

[35] Featherstone J.D.B., Doméjean S. (2012). Minimal intervention dentistry: part 1. From ‘compulsive’ restorative dentistry to rational therapeutic strategies. Br Dent J.

[36] Banerjee A., Doméjean S. (2013). The contemporary approach to tooth preservation: minimum intervention (MI) caries management in general practice. Prim Dent J.

[37] Dawson A.S., Makinson OF. (1992). Dental treatment and dental health. Part 1. A review of studies in support of a philosophy of minimum intervention dentistry. Australian Dent J.

[38] Dawson A.S., Makinson OF. (1992). Dental treatment and dental health. Part 2. An alternative philosophy and some new treatment modalities in operative dentistry. Australian Dent J.

[39] FDI World Dental Federation (2017). FDI policy statement on minimal intervention dentistry (MID) for managing dental caries: adopted by the General Assembly: September 2016, Poznan, Poland. Int Dent J.

[40] Sheiham A. (2002). Minimal intervention in dental care. Med Princ Pract.

[41] World Health Organization. Bangkok declaration: no health without oral health, Available from: https://cdn.who.int/media/docs/default-source/ncds/mnd/oral-health/bangkok-declaration-oral-health.pdf; 2024. Accessed 4 February 2026

[42] Savard G., Léger S., Trébosc M., Grosgogeat B., Doméjean S. (2025). Exploring occlusal caries management over 2 decades in France among general dental practitioners. JDR Clin Trans Res.

[43] de Moura R.C., Santos P.S., Matias P. (2023). Knowledge, attitudes, and practice of dentists on minimal intervention dentistry: a systematic review and meta-analysis. J Dent.

[44] Pitts NB. (2004). Are we ready to move from operative to non-operative/preventive treatment of dental caries in clinical practice?. Caries Res.

[45] Abuhaloob L., El-Osta A., Newton T., Rawaf S., Banerjee A. (2024). Could minimum intervention oral care (MIOC) delivery help improve patient access to NHS primary oral and dental care?. Br Dent J.

[46] Mazevet M., Tubert-Jeannin S., Doméjean S. (2020). Inadequacies between evidence-based dentistry, health policies, public funding and clinical practice: the case of cariology in a French context. Fr J Dent Med.

[47] Janusz C.B., Doan T.T., Gebremariam A. (2024 Jul). A cost-effectiveness analysis of population-level dental caries prevention strategies in US children. Acad Pediatr.

[48] Rederiene G., Bol-van den Hil E., Pajak-Lysek E., Eaton KA. (2024). The employment of dental hygienists in European countries: report of a European Dental Hygienists Federation/European Association of Dental Public Health Survey in 2021. Int J Dent Hygiene.

[49] Pillai S., Rohani K., Macdonald M.E., Al-Hamed F.S., Tikhonova S. (2024). Integration of an evidence-based caries management approach in dental education: the perspectives of dental instructors. J Dent Educ.

[50] Twetman S. (2024). Why is caries prevention in children so difficult? A narrative opinion. Int J Environ Res Public Health.

[51] Cramer C.K., Klasser G.D., Epstein J.B., Sheps SB. (2008). The Delphi process in dental research. J Evid Based Dent Pract.

[52] Samaranayake L., Phantumvanit P., General F.S., Varenne B. (2025). Bangkok declaration on oral health: a clarion call for action by all stakeholders. Int Dent J.

[53] Schulte A.G., Pitts N.B., Huysmans M.C., Splieth C., Buchalla W. (2011). European Core Curriculum in cariology for undergraduate dental students. Eur J Dent Educ.

[54] Fontana M., Guzmán-Armstrong S., Schenkel A.B. (2016). Development of a core curriculum framework in cariology for U.S. dental schools. J Dent Educ.

[55] Department of Health and Social Care. 10 year health plan for England: fit for the future. London: UK Government. Available from: https://www.gov.uk/government/publications/10-year-health-plan-for-england-fit-for-the-future; 2025. Accessed 4 February 2026

[56] Molyneux L., Banerjee A. (2024). Minimum intervention oral care – staging and grading carious lesions in clinical practice. Br Dent J.

[57] World Health Organization. WHO global strategy on oral health 2023–2030, Available from: https://www.who.int/publications/i/item/9789240090538; 2024. Accessed 4 February 2026

[58] Pillai S., Rohani K., Macdonald M.E., Al-Hamed F.S., Tikhonova S. (2024). Integration of an evidence-based caries management approach in dental education: the perspectives of dental instructors. J Dent Educ.

[59] Santamaria R.M., Fontana M., Chalas R. (2024). The core curriculum in cariology: fiction or reality? Challenges about implementation. Caries Res.

[60] Wang T., Tan J-Y, Liu X-L, Zhao I. (2023). Barriers and enablers to implementing clinical practice guidelines in primary care: an overview of systematic reviews. BMJ Open.

[61] Reddy M.S., D’Souza R.N., Webster-Cyriaque J. (2023). A call for more oral health research in primary care. JAMA.

[62] Scottish Dental Clinical Effectiveness Programme. Prevention and management of dental caries in children (3rd ed.). Available from: https://www.childcaries.sdcep.org.uk/; 2025. Accessed 10 January 2026

[63] Kennisinstituut Mondzorg (KIMO). Klinische praktijkrichtlijn mondzorg voor jeugdigen – Preventie en behandeling van cariës [Clinical practice guideline oral care for children – prevention and treatment of dental caries]. Available from: https://www.hetkimo.nl/wp-content/uploads/2021/02/2020.12.31-KPR-MvJ-preventie-en-behandeling-caries-DEF.pdf; 2020. Accessed 12 January 2026

